# Imaging Analysis of Patients With Meniere's Disease Treated With Endolymphatic Sac-Mastoid Shunt Surgery

**DOI:** 10.3389/fsurg.2021.673323

**Published:** 2022-01-12

**Authors:** Yawei Li, Yafeng Lv, Na Hu, Xiaofei Li, Haibo Wang, Daogong Zhang

**Affiliations:** ^1^Department of Otolaryngology-Head and Neck Surgery, Shandong Provincial Ear, Nose and Throat Hospital, Cheeloo College of Medicine, Shandong University, Jinan, China; ^2^Shandong Provincial Vertigo & Dizziness Medical Center, Jinan, China; ^3^Department of Radiology, Shandong Provincial Ear, Nose and Throat Hospital, Cheeloo College of Medicine, Shandong University, Jinan, China

**Keywords:** imaging, analysis, Meniere's disease, endolymphatic sac-mastoid shunt, surgery

## Abstract

**Objective:** Endolymphatic sac surgery is effective in treating intractable Meniere's disease (MD), but the underlying mechanism is still unknown. Our study investigated the mechanism by which endolymphatic sac-mastoid shunt (EMS) surgery is effective in treating MD by means of imaging.

**Methods:** The experiment included 19 patients with intractable MD who underwent 3D-fluid-attenuated inversion recovery (FLAIR) MRI with a 3-Tesla unit 6 h after intravenous administration of gadolinium, before EMS, and 2 years after the surgery. The enhanced perilymphatic space in the bilateral cochlea, vestibule, and canals was visualized and compared with that in the endolymphatic space by quantitatively scoring the scala vestibuli of the cochlea and by measuring the developing area of the vestibules quantitatively.

**Results:** Gadolinium was present in the perilymph of the inner ear in the cochlea, vestibules, and canals of all patients. At the 2-year follow-up, 14 (73.68%) patients had vertigo control. Both before and 2 years after surgery, significant differences were observed in the scala vestibuli scores and the area of vestibular perilymph between the affected and healthy sides. The scala vestibuli scores and the area of vestibular perilymph, however, did not differ when comparing them before and after surgery.

**Conclusions:** According to our results, endolymphatic hydrops was not significantly reduced by surgery. The mechanism by which EMS controls vertigo might be unrelated to the improvement in hydrops.

## Introduction

Endolymphatic hydrops was definitively diagnosed only by histopathological examination after death until Gadolinium (Gd)-enhanced inner ear MRI was introduced. Following intratympanic Gd injection, a 3-T MRI scan showed the first distinct images of endolymphatic hydrops in a patient with Meniere's disease (MD) in 2007 (1). Later, Nakashima (2) proposed a three-stage grading system for hydrops by evaluating the vestibular space and cochlea via dilated endolymphatic spaces and detected a percentage of endolymphatic hydrops that ranged from 47% (3) to ~90% (4–6) on the symptomatic side, and an elevated percentage also in the asymptomatic ear. Fukuoka also used 3D-fluid-attenuated inversion recovery (FLAIR) MRI following bilateral intratympanic Gd to semi-quantitatively evaluate endolymphatic hydrops in patients with MD (7). None of the patients who underwent this imaging method experienced any changes in hearing level or new-onset tinnitus, suggesting that this technique is a safe and powerful tool for the diagnosis of MD.

Endolymphatic sac surgery, first described by Portmann in 1927 (8), has been used to treat patients with medically refractory MD (9). Technical details of sac surgery can vary and include decompression of the sac alone, placement of a shunt between the endolymphatic sac and cerebrospinal fluid (CSF) space, and placement of a shunt between the endolymphatic sac and the mastoid air cells (endolymphatic sac-mastoid shunt; EMS). Endolymphatic sac shunt procedures became popular in the 1960s, following the success of the House's subarachnoid shunting procedure. However, the mechanism of EMS for this disease remains unclear. By comparing the imaging results before and after EMS surgery, we aimed to investigate the mechanism by which it is effective in vertigo control. Although some scholars have studied the mechanism of sac surgery by imaging, the results varied and the mechanism remained unclear.

## Materials and Methods

### Clinical Data

According to the diagnostic criteria formulated by the Classification Committee of the Bárány Society, 19 patients clinically diagnosed with unilateral Meniere's disease and admitted to the Department of Otolaryngology-Head and Neck Surgery of Shandong Provincial ENT Hospital between January 2017 and January 2018 were enrolled in our study. The selection criteria were as follows: (1) All patients had a diagnosis of unilateral definite Meniere's disease; (2) brainstem audiometry and brain magnetic resonance scans were used to rule out cerebellopontine angle tumor or other intracranial diseases; (3) middle ear disease was ruled out. The exclusion criteria were as follows: (1) Patients who had a history of allergy of Gd or pregnancy were prohibited from parting in this study; (2) Patients were excluded if they had undergone previous surgical treatment for the inner disease. The average age of the group was 47.21 ± 5.39, with nine men and 10 women. The number of vertigo spells and the hearing levels of each case is listed in Table 1. All patients had received standard medical treatment for at least 1 year but continued to experience recurrent vertigo. They agreed to undergo EMS surgery and to participate in this study, by providing written informed consent. Before the surgery and 2 years after it, all patients received intravenous Gd injection, followed by 3D-FLAIR MRI.

**Table 1:** Patient demographics, PTA (before and after surgery), and vertigo class.

			**Vertigo**	**Attack**		**PTA (dB HL)**	
**Patient no**	**Age**	**Sex**	**Pre-op**	**Post-op**	**Vertigo class**	**Pre-op**	**Post-op**
1	45	F	2	0	A	40	30
2	57	F	10	2	B	65	60
3	46	M	15	4	B	45	50
4	50	F	5	0	A	47.5	45
5	43	F	3	0	A	32.5	25
6	39	F	10	0	A	31.25	25
7	53	M	20	0	A	80	45
8	47	F	12	1	B	75	30
9	56	M	5	0	A	55	40
10	47	F	2	0	A	65	35
11	41	F	7	0	A	57.5	80
12	52	M	2	0	A	65	70
13	42	F	15	4	B	35	35
14	45	M	20	8	B	40	35
15	42	M	12	10	D	30	35
16	50	F	5	6	D	55	55
17	43	M	10	9	D	60	75
18	55	M	10	11	D	45	30
19	44	M	13	15	D	45	50

#### EMS

After complete mastoidectomy under general anesthesia, the sigmoid sinus and posterior fossa dura were skeletonized. The horizontal and posterior semi-circular canals were identified. The endolymphatic sac was identified as a thickened portion of the dura, usually deep and inferior to the posterior semicircular canal, anterior to the sigmoid sinus, and superior to the jugular bulb. The lateral aspect of the sac was incised, and a silver clip was placed into the sac, extending into the mastoid cavity.

#### Imaging Method

**Figure 1:**
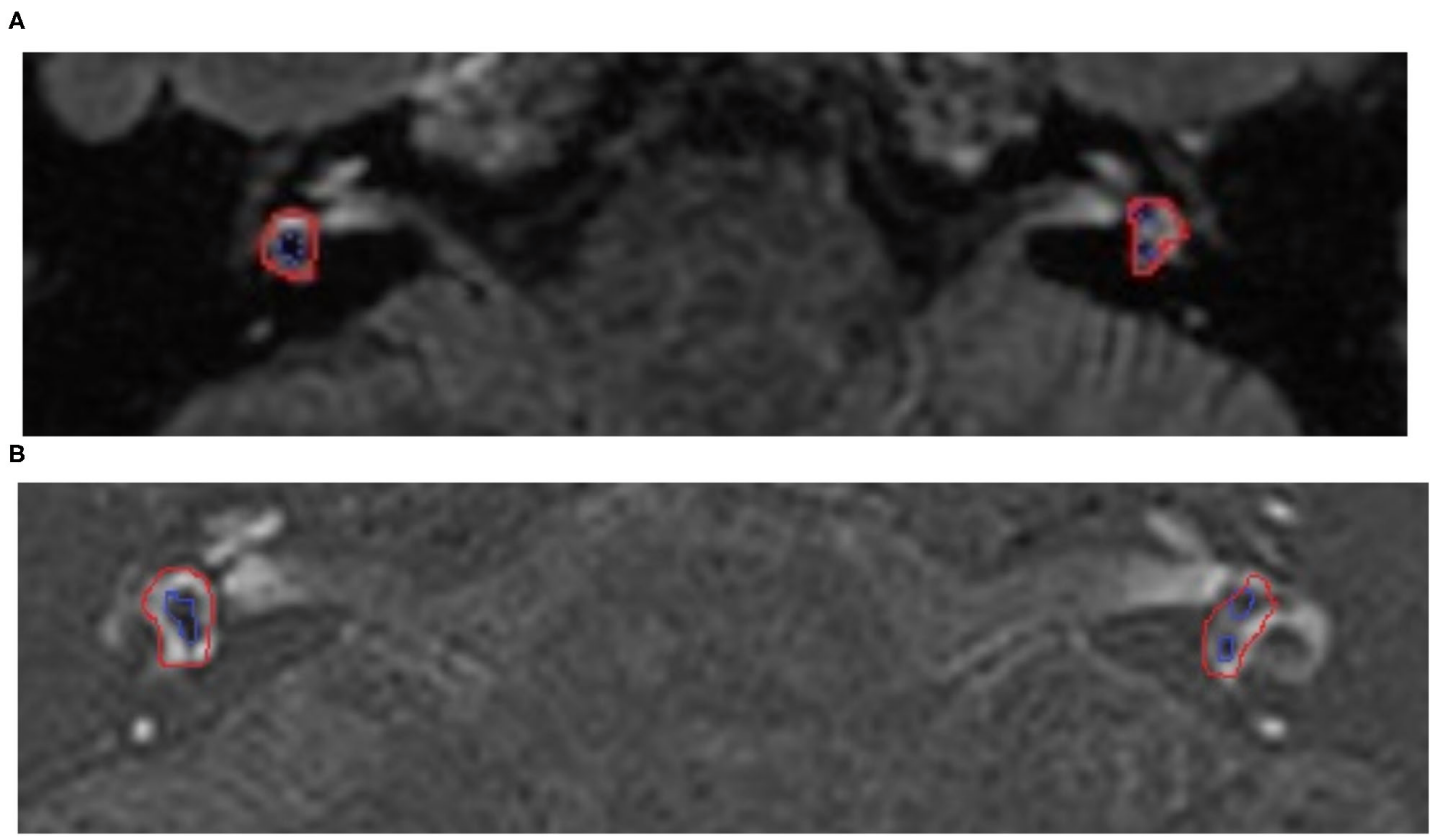
The 3D-fluid-attenuated inversion recovery (FLAIR) MRI views of a 50-year-old woman with Meniere's disease (MD) on the right side before and after surgery. The long-term control of vertigo 2 years after the operation was class A. **(A)** MRI before the operation, the red line indicated the entire vestibular area, while the blue line indicated the endolymphatic area. The low density of the vestibular on the right was larger than the left, showing vestibular hydrops on the right side. **(B)** MRI after the operation, the low density of the vestibular on the right was still larger than the left, but did not vary after surgery. This result indicated the vestibular hydrops was not alleviated before and after surgery.

A total of 19 patients underwent intravenous injection of Gd (0.4 ml/kg). We obtained 3D-FLAIR MRI scans 6 h after injection using a GE Discovery 750w 3T MRI scanner with an eight-channel phased-array coil. The parameters for 3D-FLAIR included: repetition time (TR) = 9,000 ms, echo time (TE) = 82.3 ms, inversion time (TI) = 2,500 ms, echo train length (ETL) = 140, BW = 35.7 kHz, matrix size = 256 × 256, number of excitation (NEX) = 2, slice thickness = 1.6 mm, field of view (FOV) = 20.5 cm × 17.4 cm, and a scan time of 5 min 46 s. The parameters for 3D-T2 weighted scan included: TR = 2,500 ms, TE = 102 ms, ETL = 120, BW = 41.7 kHz, matrix size = 256 × 256, NEX = 2, slice thickness = 1 mm, FOV = 20.5 cm × 17.4 cm and a scan time of 2 min 41 s. Two experienced radiologists blinded to the patient diagnosis independently reviewed the 3D-FLAIR axial findings of the bilateral inner ears of all patients. Scores for the cochlea were as follows: (1) a score of 2 indicated that the scala tympani/scala vestibuli had developed well; (2) a score of 1 indicated that the scala tympani/scala vestibuli had developed uneven or thinner; (3) a score of 0 indicated that the scala tympani/scala vestibuli was unclear or could not be visualized. Briefly, the scope of imaging was the perilymphatic area of the largest imaging section of the bilateral vestibules. Areas for the vestibule perilymph were calculated as the mean of three measurements (area of perilymph = the entire area of the vestibule – area of endolymphatic) (Figure 1). In a preliminary study, 32 patients with unilateral MD were intratympanically injected with Gd, followed by 3D-FLAIR MRI, which showed significant differences in scala vestibuli scores but not scala tympani scores, and area of vestibular perilymph on the affected and healthy sides (10). Therefore, this study measured scala vestibuli scores and area of vestibular perilymph for the purpose of grading. We reconstructed the images of 3D-FLAIR by using Volume View processing technology.

### Statistical Analysis

All statistical analyses were performed using SPSS 17 statistical software. Scala vestibuli scores were compared using the Wilcoxon signed-rank sum tests, and the area of vestibular perilymph was compared using paired sample *t*-tests. Before and after surgery, scala vestibuli scores were compared using the Mann-Whitney U test, and area of vestibular perilymph using paired sample *t*-tests. Measured values are expressed as mean ± *SD*. All statistical analyses were performed with a *P*-value < 0.05, which was considered statistically significant.

## Results

Gadolinium (Gd) was observed in almost all parts of the perilymph in the cochlea, vestibule, and canals of 19 patients. Our measuring method was based on the earlier method proposed by Nakashima in 2009 but was not identical. Before surgery, the scala vestibuli score on the affected side was 0.57 ± 0.52, and 1.66 ± 0.5 on the healthy side. On the affected side, the area of vestibular perilymph was 5.73 ± 2.99 mm^2^, while it was 8.89 ± 2.52 mm^2^ on the healthy side. The differences in scala vestibuli scores (*Z* = 3.426, *P* < 0.05) and area of vestibular perilymph (*t* = 2.65, *P* < 0.05) on the affected and healthy sides were statistically significant.

Two years after EMS, the scala vestibuli score on the affected side was 0.64 ± 0.67, and 1.64 ± 0.67 on the healthy sides. At this time, the area of vestibular perilymph on the affected side was 5.7 ± 2.89 mm^2^, while it was 8.52 ± 2.54 mm^2^ on the healthy side. The differences in scala vestibuli scores (*Z* = 2.869, *P* < 0.05) and area of vestibular perilymph (*t* = 2.39, *P* < 0.05) on the affected and healthy sides were also statistically significant. The differences in scala vestibuli scores before and after surgery on both the affected (*Z* =0.447, *P* > 0.05) and healthy (*Z* = 0.000, *P* > 0.05) sides, however, did not differ significantly. Similarly, the area of vestibular perilymph before and after surgery on both the affected (*t* = 0.74, *P* > 0.05) and healthy (*t* = 0.66, *P* > 0.05) sides did not differ significantly. The detailed data of scala vestibuli scores and area of vestibular perilymph of 19 patients before and after surgery were shown in Tables 2, 3. Among the 19 patients, nine patients (47.37%) achieved class A control, and five patients (26.32%) achieved class B control.

**Table 2:** Preoperative and postoperative 3D-FLAIR MRI comparison of the affected and healthy sides of 19 patients with Meniere's disease (*x* ± *s*).

	**Affected ear pre-op**	**Affected ear post-op**	**Unaffected ear pre-op**	**Unaffected ear post-op**
Scala vestibuli score	0.57 ± 0.52	0.64 ± 1.66	0.66 ± 0.50	1.64 ± 0.67
Area of vestibular perilymph	5.73 ± 2.99	5.70 ± 2.89	8.89 ± 2.52	8.52 ± 2.54

**Table 3:** Detailed data of 19 patients.

	**Scala vestibuli score (point)**				**Area of vestibular perilymph (mm** ^**2**^ **)**			
**Patient**	**Affected pre-op**	**Affected post-op**	**Healthy pre-op**	**Healthy post-op**	**Affected pre-op**	**Affected post-op**	**Healthy pre-op**	**Healthy post-op**
1	1	1	2	3	5.46	5.23	10.34	10.89
2	0	0	2	2	3.12	3.07	9.43	9.46
3	1	1	2	2	6.01	5.99	12.63	13.21
4	0	0	1	2	10.89	10.75	7.01	6.99
5	1	2	1	2	6.41	5.98	8.70	8.73
6	1	0	1	1	5.27	5.34	7.25	7.26
7	1	0	1	1	4.38	4.36	8.96	8.46
8	0	0	1	1	5.03	4.89	5.99	6.59
9	1	1	1	2	2.09	2.03	11.80	10.23
10	0	0	2	2	3.13	3.49	10.03	9.15
11	0	0	2	0	1.79	1.78	11.84	10.23
12	0	0	2	2	4.01	4.23	13.09	13.54
13	0	1	2	2	6.23	6.19	4.89	3.26
14	1	1	2	2	10.78	10.61	11.89	10.01
15	1	1	2	2	10.45	10.45	8.30	5.68
16	1	2	2	2	3.25	3.33	6.53	7.26
17	1	1	1	2	5.78	5.86	5.45	7.58
18	0	0	1	0	4.27	4.28	8.09	6.01
19	1	1	2	2	10.69	10.64	7.01	7.23

## Discussion

Meniere's disease (MD) is a complicated inner ear disease, and endolymphatic hydrops is thought to be the pathological basis for this condition. As we could not visually observe hydrops *in vivo*, imaging methods have been applied to detect the condition of endolymph, perilymph, and endolymphatic sac hydrops. Both 3D-FLAIR and 3D-real IR (inversion recovery) MRI can differentiate the endolymphatic space from the perilymphatic space, but the latter is not as sensitive as the former to the low density of Gd, so we used 3D-FLAIR MRI as the preferred imaging method (11, 12). However, some authors have questioned this imaging method because inversion time can affect the area of the endolymphatic space (13, 14), so different inversion times could result in differences in imaging results and accordingly affect the assessment of hydrops. Liu applied 2,350 ms as the inversion time to detect the condition of hydrops in patients with endolymphatic shunt surgery, and in this study, they found that patients who underwent shunt surgery continued to have endolymphatic hydrops (15).

The mechanism of sac surgery for vertigo control remains unclear. Gibson reported that an abnormal fluid in the inner ear caused the endolymphatic sac to secrete glycoproteins, causing drainage of the endolymph toward the sac by which vertigo is caused (16). Scholars have questioned that if hydrops can be alleviated, symptoms such as vertigo, ear fullness, or tinnitus can be controlled. However, a post-mortem histopathological study of the temporal bone of 15 patients who had undergone EMS found endolymphatic hydrops in all patients (17). Interestingly, although the endolymphatic sac was not exposed in five of these patients, four experienced postoperative control of vertigo. In the other eight patients, the endolymphatic sac was exposed, but the drainage tube was not embedded in the endolymphatic sac cavity; of these eight patients, four showed postoperative control of vertigo. In only two patients, the endolymphatic sac was exposed, and the drainage tube was embedded in the cavity of the endolymphatic sac, but neither of them showed postoperative vertigo control. Therefore, the control of vertigo was not related to the condition of the endolymphatic sac. This procedure may only reinforce the vertigo threshold. In 1977, Torok suggested that the placebo effect might be a factor in vertigo control (18). Thus, in conclusion, endolymphatic sac surgery was found to not control vertigo by relieving hydrops.

Following the changes in imaging parameters before and after EMS surgery, we found that the mechanism of vertigo control was not due to the improvement in hydrops. In 2013, Uno et al. reported changes in endolymphatic hydrops after endolymph sac surgery, which were observed with 3D-FLAIR MRI after intratympanic injection of Gd. Although only 28.6% of patients had negative hydrops after sac surgery, all patients had vertigo suppression. However, in 2014, Liu (19) found that endolymphatic hydrops in the cochlea and vestibule improved 3 months after endolymphatic sac decompression, which is contrary to our results. This result might have been due to the different surgical approaches and observation times. Pender performed a meta-analysis of MD to demonstrate that endolymphatic hydrops began in the cochlea, followed by the saccule, utricle, ampullae, and finally affected the semi-circular canals (20). This finding might have affected our results because we chose the timepoints as before surgery and 2 years after surgery for comparison. Moreover, the extent of hydrops was reported to be unrelated to the surgical approach or the condition of sac exposure, confirming that endolymphatic sac surgery could not relieve hydrops but could control vertigo (18).

In conclusion, our findings indicate that the mechanism of EMS surgery does not involve the improvement of hydrops. In the future, the further examination must be conducted in clinics to confirm the relationship between endolymphatic hydrops and vertigo control, and to provide new theories for the mechanism of MD. As for the potential of surgery to affect hydrops, a large number of cases with or without surgery should be accumulated, and further comparisons should be made.

## Data Availability Statement

The original contributions presented in the study are included in the article/supplementary material, further inquiries can be directed to the corresponding author/s.

## Ethics Statement

Written informed consent was obtained from the individual(s) for the publication of any potentially identifiable images or data included in this article.

## Author Contributions

YLi contributed to the conception of the work. YLv and XL contributed to the surgery. YLi collected data and performed the analysis. NH contributed to the part of imaging. HW and DZ contributed to the study design. All authors contributed to the article and approved the submitted version.

## Funding

This work was supported by Shandong Provincial Natural Science Foundation (No. ZR2020MH179) and Taishan Scholars Program of Shandong Province (No. ts20130913).

## Conflict of Interest

The authors declare that the research was conducted in the absence of any commercial or financial relationships that could be construed as a potential conflict of interest.

## Publisher's Note

All claims expressed in this article are solely those of the authors and do not necessarily represent those of their affiliated organizations, or those of the publisher, the editors and the reviewers. Any product that may be evaluated in this article, or claim that may be made by its manufacturer, is not guaranteed or endorsed by the publisher.
